# Cough up your lungs

**DOI:** 10.1007/s12471-018-1116-2

**Published:** 2018-04-17

**Authors:** L. A. J. Rammeloo, N. A. Blom

**Affiliations:** 1 Department Paediatric Cardiology, VU University Medical Center, Amsterdam, The Netherlands; 2 Department of Paediatric Cardiology, Leiden University Medical Center, Leiden, The Netherlands

## Answer

### Plastic bronchitis, expectoration of an airway cast

This rubbery bronchial cast is composed of proteinaceous and chylous material deposited from lymphatic fluid into the airways due to high intrathoracic lymphatic pressures.

This large branching cast of the airways was expectorated by a 6-year-old patient, almost 2 years after palliation of tricuspid atresia with a total cavopulmonary connection (Fontan circulation).

The formation of these casts, also known as plastic bronchitis, is a rare condition and almost always associated with underlying disease. In cardiac conditions, those with high venous and high lymphatic pressures are suspected [1].

Bronchial casts can sometimes fill the large parts of the airways, and present as an acute, life-threatening emergency.

There are several treatment options with varying degrees of success. Treatment of the underlying cardiac conditions can resolve the formation of these casts. Interventional closure of leaking intrathoracic lymph vessels is a promising treatment option in patients after palliative treatment of the heart defect with residual high venous and lymphatic pressures [2].

## References

[1] Eberlein MH, Drummond MB, Haponik EF (2008). Plastic bronchitis: a management challenge. Am J Med Sci.

[2] Dori Y, Keller MS, Rome JJ (2016). Percutaneous lymphatic embolization of abnormal pulmonary lymphatic flow as treatment of plastic bronchitis in patients with congenital heart disease. Circulation.

